# Ribosomes: from conserved origin to functional/medical mobility and heterogeneity

**DOI:** 10.1098/rstb.2023.0393

**Published:** 2025-03-06

**Authors:** Andre Rivalta, Disha-Gajanan Hiregange, Tanaya Bose, K. Shanmugha Rajan, Ada Yonath, Ella Zimmerman, Miriam Waghalter, Gil Fridkin, Irene Martinez-Roman, Liat Rosenfield, Aliza Fedorenko, Anat Bashan, Hagith Yonath

**Affiliations:** ^1^ Department of Chemical and Structural Biology, Weizmann Institute of Science, Rehovot, Israel; ^2^ Department of Organic Chemistry, Israel Institute for Biological Research, Ness Ziona, Israel; ^3^ Human Genetics Institute and Internal Medicine A, Sheba Medical Center, Ramat-Gan and Tel-Aviv University, Tel Aviv, Israel

**Keywords:** ribosome heterogeneity, ribosome mobility, protoribosome, ribosomopathies, genetic diseases, ribosomal mutations

## Abstract

Ribosomes, the molecular machines that translate the genetic code from mRNA into proteins in all living cells, are highly structurally conserved across all domains of life and hence are believed to have evolved from a structurally unified pocket. Initially perceived as uniform cellular factories for protein synthesis, currently, ribosomes have emerged as more complex entities. Structural, medical and biochemical studies, including ours, have revealed significant variability in their compositions across tissues, species, functions and developmental stages, highlighting their multifunctional potential. Moreover, the diversity of ribosomes, their components and their associated biological factors challenge the traditional perception of uniform interactions under various conditions, including stress, and expose their mobility and heterogeneity. Evidence for their functional diversity can be seen even in modifications of ribosomal genes, where minor changes may play critical roles under stress or may lead to diseases called ribosomopathies, including Diamond–Blackfan anaemia, some types of cancer and Alzheimer’s disease. Thus, through in-depth structural explorations, we improve the understanding of the mechanisms regulating protein biosynthesis in response to various environmental stressors. These findings should potentially reshape the perceptions of the various ribosomal roles.

This article is part of the discussion meeting issue ‘Ribosome diversity and its impact on protein synthesis, development and disease’.

## 1. Introduction

**Figure 1.**
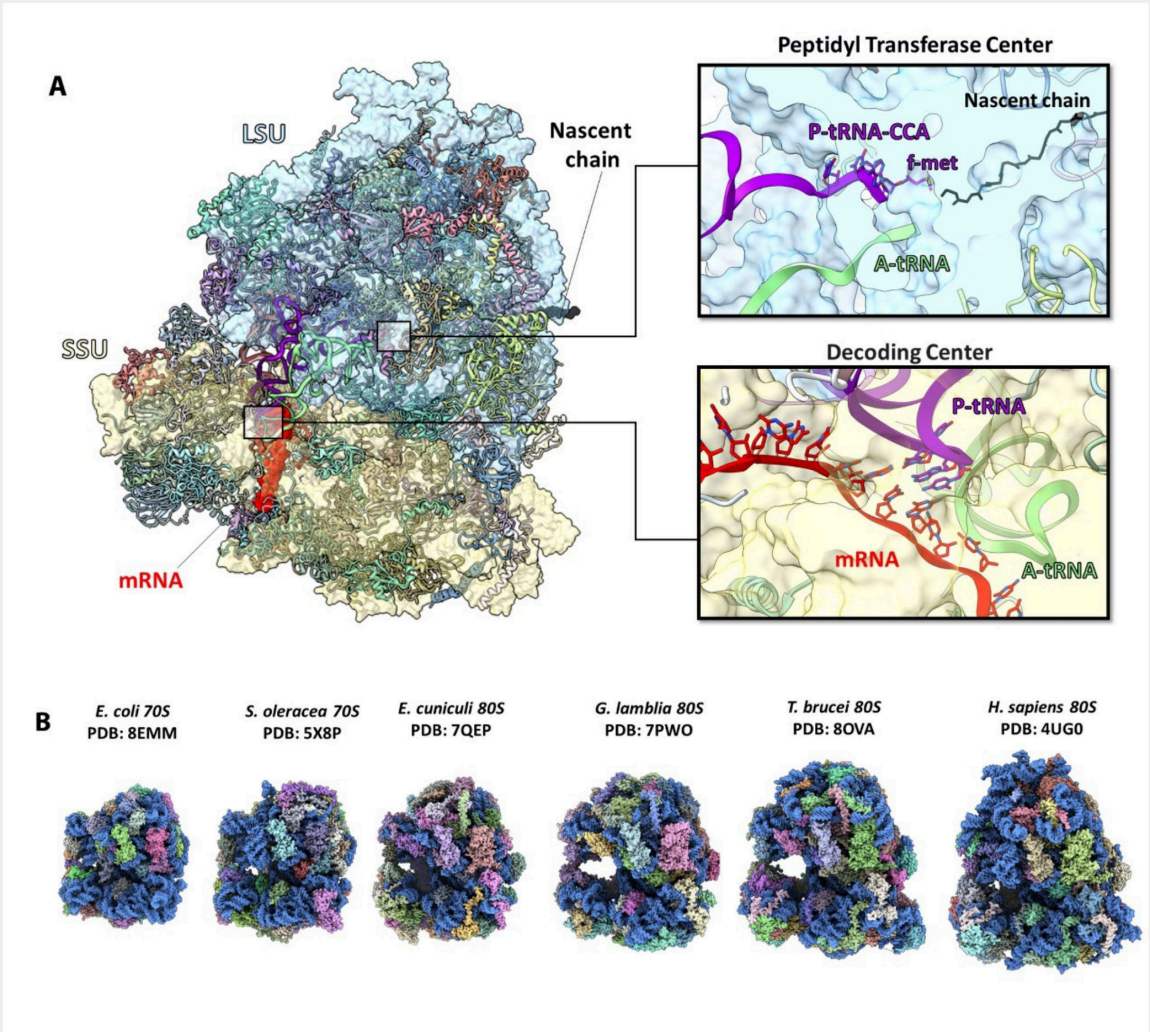
Functional centres of the ribosome (*A*) The space-filling model of a ribosome with LSU-rRNA, SSU-rRNA mRNA, P-tRNA, A-tRNA and nascent chain shown in sky blue, yellow, red, purple, green and black, respectively. Proteins are shown in ribbons in different colours. Zoomed-in images show the peptidyl transfer centre (top right panel) and decoding centre (bottom right panel). (*B*) Atomic structures of ribosomes from different prokaryotic and eukaryotic organisms. While ribosomes may differ in size and the number of their components, they all share a fundamental core structure. rRNA is shown as a space-filling model coloured in blue, whereas ribosomal proteins are highlighted in different colours.

Ribosomes, irrespective of their cellular origins—namely eukaryotes or prokaryotes (including archaea and bacteria)—are the universal cellular particles, composed of ribosomal proteins (rProteins) and ribosomal RNA (rRNA), which execute two essential and interrelated functions across all cells: decoding the genetic code according to the sequences encoded in the mRNA chains and catalyzing peptide bond formation between activated amino acids bound to the CCA ends of tRNA molecules, which are accommodated sequentially according to the genetic code [1,2] (figure 1). These universally common tasks occur even in the absence of a nuclear envelope in prokaryotic cells, or in the presence of a nucleus in eukaryotic cells, where the genetic material is segregated from the cytoplasm. It was found that although historically ribosomes were assumed to be invariable, some differences in their performance, in health as well as diseases that are linked to genetic mutations, have been observed [3]. Variations in the standard mechanisms of the mRNA translation arise mainly in response to abnormalities, to additional cellular requirements or to other challenges associated with maintaining proper functionality. From the structural evolution perspective, ribosomes can be described as particles with a conserved origin and main function, which diverged into entities with somewhat varied compositions or modified particles with enriched or depleted operational capacities, compared to their ancestral forms. These also include medically challenging developments, such as human ribosomes being implicated in genetic diseases known as ribosomopathies—namely a collection of disorders in which clinical syndromes result from genetic abnormalities that cause impaired ribosome biogenesis and/or depletion of the amount of functional ribosomes. Such mutations may be germline or somatic and can be associated with different diseases, such as some cancers.

## 2. The protoribosome

Among the fully preserved ribosomal tasks is the formation of peptide bonds. All ribosomes are built of two subunits: the small subunit accommodates the mRNA chain to be translated, and the large one houses the peptidyl transferase centre (PTC) site, which is responsible for peptide bond formation (figure 1). *In vivo*, ribosomes act constantly, capable of catalyzing the formation of up to 20 peptide bonds per second in *Escherichia coli* [4], with a remarkably low error rate (about 1:10 000 in *E. coli* and down to 1:1 00 000 in yeast) [5,6]. Interestingly, although all ribosomes execute their primary task—decoding the genetic information into proteins, nearly identically—certain ribosomal components may also be involved in additional functions, dictated by specific cellular needs, which are not explored in detail here. Nevertheless, despite the diversity and heterogeneity (described below), the main catalytic region of the ribosomes, namely the PTC, is the ribosomal region that shows structural (but not sequence) conservation and seems to be the kernel around which the ribosomes evolved.

According to the ‘RNA world’ hypothesis, RNA played a central role in the prebiotic era. In agreement with this idea, it is reasonable to propose that the rRNA-made PTC was the origin of the ribosome. Already in 1968, Francis Crick suggested that the original ribosome could have been made entirely from RNA [7]. Since then, the idea of an ‘RNA world’ preceding the emergence of protein-based machinery has only gained traction, also owing to the pioneer structural studies that showed that even in the modern ribosome the PTC is entirely made of RNA [1,2], thus indicating its enzymatic capabilities. A recent study showcasing the development of an RNA-replicating enzyme with RNA-cleaving activity further reinforces the plausibility of an RNA-based prebiotic world [8].

**Figure 2.**
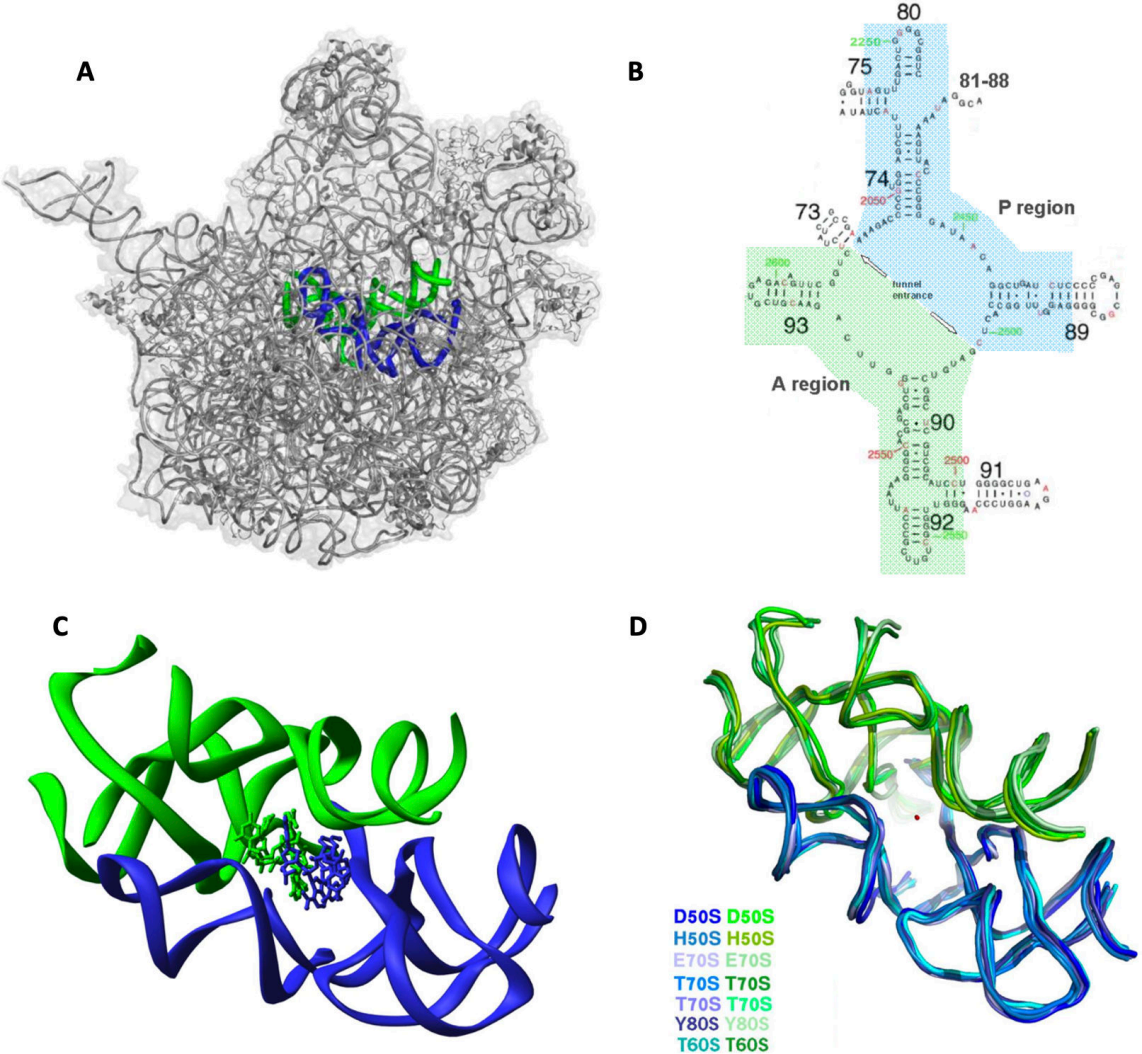
The conserved rRNA pocket corresponding to the protoribosome. In blue, is the A-site subregion, in green the P-site subregion. (*A*) The location of the protoribosome core within the contemporary ribosome (in grey). (*B*) Two-dimensional structure diagram of the semi-symmetrical pocket (*E. coli* numbering in red, *Deinococcus radiodurans* numbering in green; rRNA helix numbers are shown in black). (*C*) The semi-symmetrical structure formed by the A and P regions that accommodate the 3′ ends of the two A- and P-site tRNA molecules. (*D*) The high three-dimensional structural conservation of the rRNA region that we hypothesized as the protoribosome, across domains and species (overlay of the region within *D. radiodurans* (PDBID: 1NKW), *Haloarcula marismortui* (PDBID: 4V9F), *E. coli* (PDBID: 4V4Q), *Thermus thermophilus* (PDBIDs: 4V4I, 4V51), yeast (PDBID: 4V7R), *Tetrahymena thermophila* (PDBID: 4V8P)).

A region composed of up to 180 nucleotides surrounding the contemporary PTC exhibits remarkable structural semi-symmetry within the otherwise largely asymmetric ribosome (figure 2). Such internal symmetry may be a remnant of the dimerization of two RNA monomers [9]. In the prebiotic era, we hypothesize that two rather short RNA strands could occasionally interact and form pocket-like bodies capable of hosting two amino acids, either by themselves or activated and bound to short RNA chains. These components could interact with each other and establish the formation of peptide bonds, which could have provided a new reaction centre to following similar reactions, thus forming growing nascent peptide chains. Such PTC-like precursor of the ribosome could have evolved into a machinery capable of forming additional bonds by the amino acids brought to it, hence translating the genetic code while protecting the growing polypeptides.

To test, demonstrate and strengthen this hypothesis, we constructed several RNA chains capable of (i) forming pockets by dimerizing within laboratory settings and (ii) mimicking the peptidyl transferase reaction between amino acids within the contemporary ribosomes. Thus, within the proto-RNA complex, just about 70−180 nucleotides, with the proper sequence for the formation of molecular pockets capable of establishing a peptide bond, namely a PTC-like arrangement, allowed us to detect the PTC-like action. This experimental evidence confirms the plausibility of RNA dimers functioning as scaffolds for peptide bond formation, supporting the notion that such RNA structures could have existed and facilitated the pre-life early events of protein synthesis.

Based on their pocket-like structure and mode of operation we called these pockets protoribosomes [10,11]. Obtaining chemically active protoribosomes verified our suggestion about the existence of prebiotic active pockets, similar to those found within contemporary ribosomes. Furthermore, our recent studies indicate that occasional events could lead to the formation of chemically active structures resembling the current PTC, namely RNA dimers that could attract activated amino acids, i.e. when bound to short RNA chains like tRNA or even only the CCA ends of the tRNAs.

Through accretion, over time, the protoribosomes evolved to larger multi-component cellular machines with increased complexity and more sophisticated mode of operation, by the addition of rProteins and extended segments of rRNA, while maintaining the core’s structure [12,13].

## 3. The tRNA E-site has functional mobility

Although only two tRNA sites are essential for the formation of peptide bonds, the elongation step of protein biosynthesis involves the sequential binding of tRNAs to three distinct sites within the ribosome, called the A- (aminoacyl site), P- (peptidyl site) and E-sites (exit site). While the necessity of the A- and P-sites for the primary chemical ribosomal function became apparent early in global research, the E-site was discovered only in 1981 [14], long after the existence of the A- and P-sites was established. Its existence was verified once the structure of the large ribosomal subunit was determined [1,2]. The functional importance of the after-duty exiting tRNA was suggested much later [15], once it was shown that the integrity of the G2421-C2395 (*E. coli* numbering system) base pair in the ribosomal E-site is crucial for protein synthesis [16]. Furthermore, it was established that the rRNA helix 68, which is close to the E-site outer edge, can either impede or facilitate elongation, owing to its functional mobility [17].

## 4. Ribosomal functional heterogeneity and mobility in prokaryotes

The concept of heterogeneous ribosomes was introduced in the late 1950s and 1960s [18]. However, due to experimental limitations and poor knowledge of the structure of the ribosomes at that time, it was difficult to determine whether this observed heterogeneity was simply an artefact or had a functional role. As discussed in a review by Genuth & Barna [3], researchers even considered the possibility that a single type of ribosome could translate a single protein type, before the concept of uniformly translating ribosomes became widely accepted.

In the following decades, several studies suggested that prokaryotes’ environmental or metabolic alterations, including stress, could induce variability in ribosome composition and function modes. Such changes span from modifications of rProteins or rRNA to altered rProtein stoichiometry, as well as the incorporation of protein variants (e.g. paralogs) [19].

A prominent example of ribosome variations under stress conditions is the response to antibiotics. A well-known rRNA modification is the mono- or di-methylation of nucleotide A2058 (*E. coli* numbering), carried out by a methyltransferase belonging to the *Erm* family. Given that such modification negatively impacts translation [20], the gene expressing this enzyme is often inducible and is only expressed in the presence of macrolide antibiotics [21].

In addition, our structural studies highlighted how ribosomal protein uL22 plays a crucial role in modulating the shape of the protein exit tunnel and conferring resistance to macrolide antibiotics like erythromycin. Mutations in the β-hairpin loop of uL22, located away from the drug binding site, can alter the tunnel’s shape and create a wider path for nascent proteins, bypassing the antibiotic barrier [22,23].

## 5. Ribosomal heterogeneity and mobility in eukaryotes, linked to diseases and ageing

### (a) rRNA modifications

Both species-specific and conserved rRNA modifications exist in ribosomes of all organisms studied to date, but eukaryotes have a higher rate of post-transcriptionally modified residues [24]. This high incidence of modifications is a critical contributor to ribosome heterogeneity and potentially more fine-tuned translational control [25], as outlined below.

**Figure 3.**
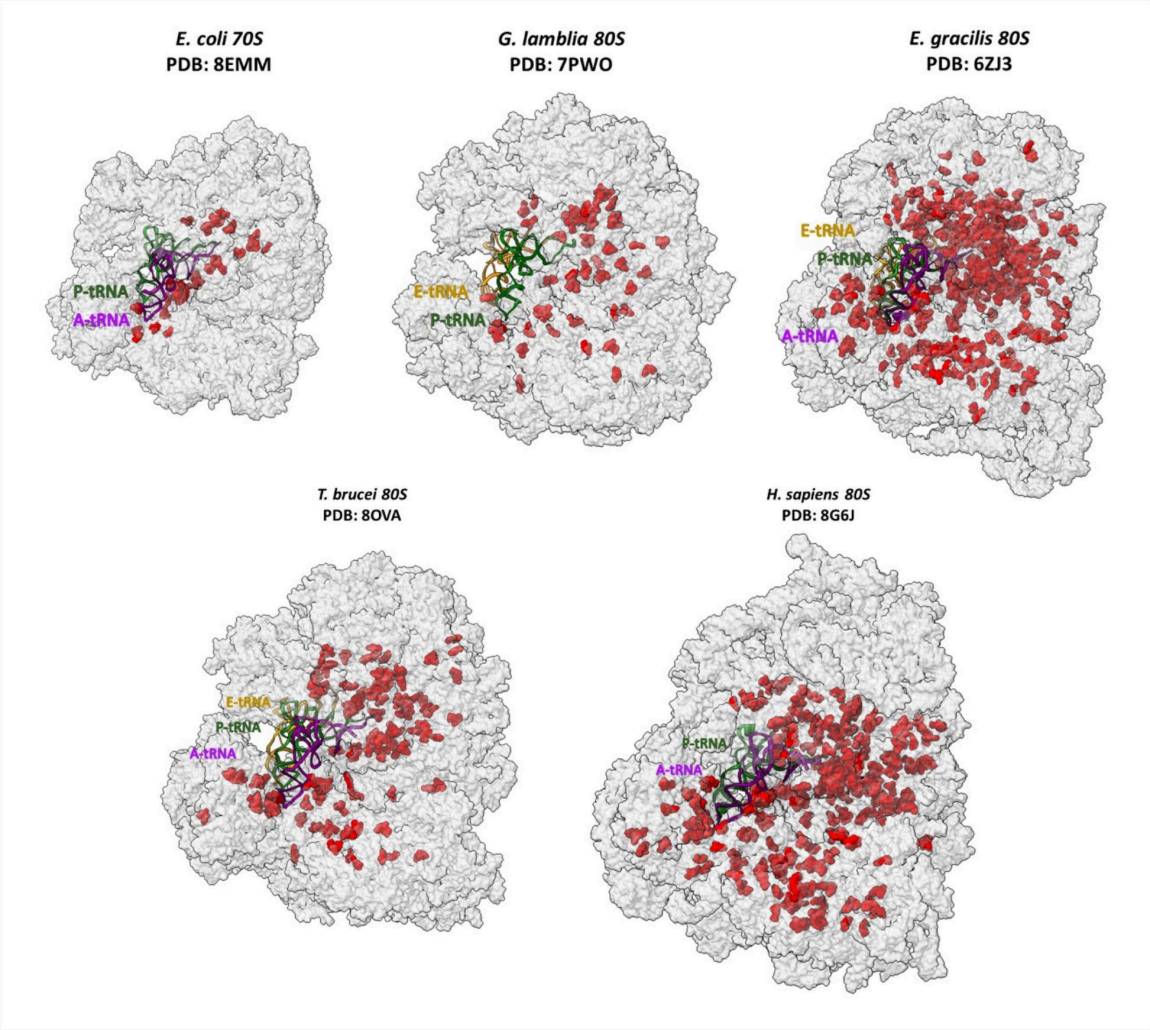
rRNA modifications across ribosome structures from evolutionary different organisms. Space-filling models of ribosomes from different organisms are shown in grey, with tRNA molecules displayed in ribbon representation using different colours (P-site in green, A-site in purple, E-site in mustard). rRNA modifications, highlighted in red, can be seen mainly clustered around key functional centres but also spread in other sites of the ribosome.

Although many studies describing ribosome heterogeneity deal with the composition of their rRNA nucleotides or their rProteins, the functional roles of post-transcriptional rRNA modifications are only starting to come into focus, from physiological processes such as ribosome biogenesis [26,27] to aberrant phenomena, including cancer [28–30]. These rRNA modifications were earlier thought to be static in ribosomes, but recent quantitative approaches suggest that they are highly dynamic throughout the developmental cycle of cells [31–33], as well as altering between different disease conditions, such as cancer [34,35] and viral infections [36]. RNA modifications can be detected using a range of techniques, often specific for the relevant modification, or useful for quantification [28,29,37]. Thanks to improved resolution, cryo-electron-microscopy (EM) studies can be used as an additional technique that effectively visualizes the presence of rRNA modifications in ribosomes across almost all kingdoms of life (figure 3). Modifications can be found in the base or the sugar of a nucleotide: cytidine can be acetylated, and methyl groups can be added to any base, but the most common modifications are the 2′-*O*-methylation of the sugar and the conversion of uridine to pseudouridine [28,38]. Pseudouridine, the first modified RNA residue to be found, is an isomer of uridine with additional H-bond capabilities, and in rRNA, it is located mainly around key functional regions [39,40]. A cryo-EM study comparing fully modified and pseudouridine-free ribosomes in yeast demonstrated that pseudouridines are required to stabilize the key regions crucial for correct inter-subunit dynamics [41]. Among species, the locations and numbers of pseudouridines on rRNA vary: 10 pseudouridines are found in *E. coli*, whereas the yeast ribosome includes 45, humans include 104 [25] and *Trypanosoma brucei*, a parasitic eukaryote, includes 70 pseudouridines [31]. Also, quantitative mass-spectrometry analysis of rRNA from yeast, *T. brucei* and human cells suggested that not all rRNA were equally modified within cells [31,42,43]. Broadly, pseudouridine modifications play essential roles in maintaining ribosomal function and cellular physiology. The conversion of uridines to pseudouridines is accomplished by evolutionarily conserved ribonucleoprotein complexes made of small nucleolar RNAs (snoRNA), called H/ACA snoRNA, and multiple proteins, including the catalytic subunit pseudouridine synthase, i.e. the human dyskerin and its homologs [44,45]. Studies in yeast showed that the absence of specific pseudouridines in functional domains of rRNA using H/ACA snoRNA mutants may lead to defects in cell growth, rRNA processing and other ribosome functions [46,47]. Similarly, defects in translation were observed in yeast and human cells carrying a catalytically inactive pseudouridine synthase (encoded by the *Cbf5* and *DKC1* genes in yeast and mammals, respectively) [48]. Interestingly, dyskerin’s pseudouridine synthase activity was found to be vital for haematopoietic stem cell differentiation, as observed in X-linked dyskeratosis congenita patients, who carry mutations in *DKC1* [49]. Defective rRNA pseudouridylation due to altered dyskerin mRNA expression was also found to be linked to human breast carcinoma [50,51].

Our recent studies suggest that rRNA pseudouridylation is developmentally regulated in mammalian parasites such as *T. brucei* and *Leishmania* species and that the loss of even a single modification in functional domains of rRNA may disrupt ribosomal function [31,52]. For instance, in *T. brucei*, a missing pseudouridine altered the stoichiometry of ribosomal proteins and contributed to translation defects of a subset of mRNAs [31]. In *Leishmania*, the absence of a conserved pseudouridine in helix 69 of the large ribosomal subunit impaired the selective accommodation of specific tRNAs in the A-site, affecting the translation of mRNAs carrying codon bias [52]. It remains unclear how rRNA modifications in distal regions from the functional domains contribute to the catalytic activity of the ribosome and if these modifications have stage- or species-specific functions. Recent studies suggest that snoRNAs may also perform additional functions on the translating ribosomes that are independent of their role in guiding rRNA modifications [53]. This suggest that not all phenotypes observed during ablation of snoRNA or *DKC1* arise solely due to loss of pseudouridine(s). Indeed, studies using mutated snR10 snoRNA in yeast cells showed that the pseudouridine guided by this snoRNA is not essential for cell growth and rRNA processing. Instead, a secondary sequence element on the snoRNA is essential for its chaperon-like activity, facilitating the pre-rRNA folding [54].

The 2′-*O*-methylation of ribose can happen on any nucleotide and, as mentioned, is the most frequent rRNA modification together with pseudorudine and is thought to improve helix stability by increasing base-stacking [27]. Similarly to pseudouridines, most 2′-*O*-methylations in the rRNA are carried out by a ribonucleoprotein complex, with fibrillarin (FBL) or its conserved homologs acting as the catalytic methyltransferase [55–57]. FBL is required for the pre-rRNA processing, since the modification occurs co-transcriptionally [58–61], and such modifications may be essential for correct ribosome assembly [62], not only in eukaryotes [63]. Despite lingering doubts about experimental artefacts, recent advancements in techniques strongly suggest that, in many cases, this modification varies depending on the developmental or pathological state of the cells [60]. For example, a study showed that in early development the patterns of 2′-*O*-methylation are specific for a germ layer and that in neurogenesis specifically the ribosomes differing in their 2′-*O*-methylation may contribute to establishing tissue identity. Moreover, in the same study, ablation of a modification on a specific site was found to promote stem cell differentiation toward nervous system development [64]. Similarly, the loss of a single 2′-*O*-methylation in human cells was found to lead to defects in mRNAs carrying specific codon bias [65]. Additionally, a mutation of nucleophosmin, a multifunctional chaperone involved in the regulation of 2′-*O*-methylation levels of rRNA, has been associated with dysfunctional haematopoietic stem cells as well as bone marrow failure in dyskeratosis congenita [66,67].

Given the crucial role of FBL in catalyzing 2′-*O*-methylation, rRNA processing and ribosome biogenesis, research also highlighted its importance in disease scenarios, particularly cancer [61,68]. Tumour progression relies on heightened ribosome production, which requires increased rRNA synthesis [69]. Also, it was shown that the inactivation of p53 is associated with increased FBL expression levels, leading to altered rRNA modifications and reduced fidelity in mRNA translation [70].

Although these studies have described the functional role of some single rRNA modifications or single snoRNAs in gene-specific translation, the mechanistic insights detailing how these modifications contribute to ribosome function, as well as disease phenotypes, still remain elusive.

### (b) Ribosomopathies

Many examples of heterogeneity in eukaryotic ribosomes are connected to ribosomopathies, a diverse collection of diseases that are often tissue-specific and lead to specific clinical phenotypes [71–73]. These diseases are thought to primarily arise from impaired ribosome biogenesis and/or disease-connected modified ribosomal functions. They are associated with germline or somatic mutations in the ribosomal protein genes or ribosomal biogenesis components. Some examples of ribosomopathies include Diamond–Blackfan anaemia (DBA), characterized by mutations in various ribosomal protein genes; Schwachman–Diamond syndrome, in which mutations in the *SBDS* gene, connected to ribosome biogenesis and mRNA processing, are connected to about 90% of the patients, although their precise role is unclear; cartilage hair hypoplasia, associated with the *RMRP* gene, required for the maturation of the 5.8S rRNA; and Treacher–Collins syndrome, resulting from mutations in the gene encoding the treacle protein, a key factor for rRNA transcription [71,73–75]. While X-linked dyskeratosis congenita is often considered a ribosomopathy, mainly because of mutations in the dyskerin gene, one study suggested that the disease phenotype could be associated with increased levels of apoptosis rather than ribosome biogenesis [76]. Another study was unable to find decreased pseudouridylation or impaired rRNA processing in *DKC1* mutants, instead highlighting defective telomere maintenance as a key factor [77]. Mutations in ribosomal protein genes and malfunction of ribosomal components have also been found to be associated with other diseases, such as Alzheimer’s disease [78] and several types of cancer [74].

Despite the frequency of these mutations, their structural consequences have been largely overlooked. This neglect may be due to the common opinion that mutations in ribosomal components interfere with the assembly of functional ribosomes, hence leading to a deficiency of translating ribosomes, which triggers a loss in the cell’s translational ability [73]. Indeed, for most known cases, it is tempting to suggest that the mutations may cause structural alterations of the ribosomal proteins, which prevent their incorporation into the precursor ribosome subunits. In such cases, this hinders ribosome assembly and causes a severe reduction in the number of functioning ribosomes [73,79]. However, this prevailing viewpoint seems to conflict with the observed clinical diversity: recent studies suggest explicit connections between specific mutations and the onset or progression of specific diseases, implying that, in some cases, ribosomal dysfunction is a consequence of the mutated ribosomes themselves, not only the ribosome scarcity. For example, in DBA, mutations in *RPL5* and *RPL11* were found to be associated with specific clinical phenotypes such as cleft palate and abnormal thumbs, hence, it was suggested that these rProteins fulfil specific roles in rRNA maturation or in the formation of well-functioning ribosomes [80]. In the nucleus, ribosomal proteins RPL5 and RPL11 form a complex with 5S rRNA (5S RNP), a crucial step for the integration of preribosomal particles [81]. Impaired ribosome biogenesis leads to the accumulation of the 5S RNP complex, which interacts with MDM2 and disrupts p53 homeostasis [82,83]. Additionally, the 5S RNP–MDM2 interaction has been linked to RPS19 deficiency, emphasizing its role as a key driver of DBA pathogenesis [84]. This challenges the conventional perspectives and implies the possibility of more direct involvement of mutated ribosomal proteins in cellular regulation.

Owing to these still open issues, we are trying to identify the links between particular ribosomal mutations and the type of their associated disease, alongside confirming the existence of ribosome-incorporated mutated rProteins and/or rRNA. An example is the protein encoded by the *RSP19* gene, the first and most common ribosomal protein gene that is implicated in human diseases [75,85,86]. In patients with DBA, many different mutations were identified in this gene, impacting both ribosome functions and biogenesis [75], which may be connected to the fact that, under normal physiological conditions, its protein product eS19 is exposed on the ribosome’s surface. While it remains to be proven, owing to the surface exposure of this protein, if its mutated version is expressed and incorporated, it could potentially exhibit structural changes that alter its positioning during assembly or affect the outer ribosomal structure, and consequently interfere with cellular biogenesis [75].

### (c) Ageing

Another life process associated with mutated rProteins, altered post-synthesis modifications and impaired ribosome biogenesis is ageing, which is marked by the progressive decline in tissue and organ functions in older individuals [87,88]. One of the mechanisms associated with ageing might be caused by the disruptions in the translation machinery, particularly affecting protein biosynthesis and co-translational protein folding, leading to cellular damage accumulation, which are key factors and contributors to ageing and the onset of age-related diseases [89,90]. Indeed, ageing is a complex condition encompassing several medical (or physiological) aspects, often linked to perturbations in genes involved in housekeeping functions, such as ribosome biogenesis [91]. In a syndrome resembling accelerated ageing, Hutchinson–Gilford progeria, a hallmark characteristic was increased ribosome biogenesis and translation, which may be attributed to excessive rRNA transcription, elevated rProtein synthesis and assembly of actively translating polysomes [92]. However, how alterations in ribosome biogenesis can result in specific medical defects is still not fully understood.

## 6. Conclusions and future plans: taking advantage of mutations causing surface heterogeneity

The highly efficient, complex and diverse machinery represented by the functional ribosomes is the culmination of billions of years of evolution, which brought the primordial RNA-based structures, as described by the protoribosome proposition, to acquire additional components, such as ribosomal proteins and extended rRNA segments, which enhanced stability and functionality. This accretionary evolution preserved the PTC as a conserved core responsible for producing proteins, which are the main products of the ribosomes, while enabling the ribosomes to adjust themselves to diverse cellular demands. Variations in rRNA and rProtein composition, as well as post-transcriptional modifications like pseudouridylation and 2′-*O*-methylation, highlight the ribosome’s dynamic capacity for fine-tuning translation, enabling organisms to adapt to physiological and environmental challenges. These modifications and variations have critical implications, not only for normal cellular functions but also in the context of diseases, including ribosomopathies and cancers, as well as ageing.

Like many others, by delving into the complexity of ribosomal structure, we have uncovered intricate details that yielded more understanding of ribosomes’ function across diverse organisms. These insights illuminated novel mechanisms governing the regulation of protein synthesis, impacting both health and disease states. Additionally, these studies have elucidated the role of ribosomes in cellular responses to environmental stresses. However, how alterations in ribosome biogenesis can result in specific medical defects is still not fully understood. Therefore, ongoing research is increasingly focused on how mutations or altered modifications in ribosomal components contribute to diseases such as ribosomopathies and cancer. Current investigations in our lab aim at confirming or excluding the incorporation of mutated rProteins or rRNA into the ribosomes.

Another mechanism might be rRNA modifications: the snoRNAs involved in the ribonucleoprotein complexes that drive these modifications could serve as promising therapeutic targets. Nevertheless, the multifaceted roles of these small RNA molecules must be considered, adding a layer of complexity to their potential as therapeutic interventions [93,94]. Elucidating the pathological roles of ribosomal defects may pave the way for innovative therapeutic interventions or diagnostic tools targeting ribosome-related diseases and dysfunctions.

## Data Availability

This article has no additional data.
